# Mechanistic Understanding of the Effects of Pectin on In Vivo Starch Digestion: A Review

**DOI:** 10.3390/nu14235107

**Published:** 2022-12-01

**Authors:** Yeming Bai, Robert G. Gilbert

**Affiliations:** 1 Jiangsu Key Laboratory of Crop Genetics and Physiology, College of Agriculture, Yangzhou University, Yangzhou 225009, China; 2 Jiangsu Co-Innovation Center for Modern Production Technology of Grain Crops, Yangzhou University, Yangzhou 225009, China; 3 Centre for Nutrition and Food Sciences, Queensland Alliance for Agriculture and Food Innovation, The University of Queensland, Brisbane, QLD 4072, Australia

**Keywords:** pectin, starch, glycemic response, digestion, diabetes, dietary fiber

## Abstract

Obesity and type II diabetes are closely related to the rapid digestion of starch. Starch is the major food-energy source for most humans, and thus knowledge about the regulation of starch digestion can contribute to prevention and improved treatment of carbohydrate metabolic disorders such as diabetes. Pectins are plant polysaccharides with complex molecular structures and ubiquitous presence in food, and have diverse effects on starch digestion. Pectins can favorably regulate in vivo starch digestion and blood glucose level responses, and these effects are attributed to several reasons: increasing the viscosity of digesta, inhibiting amylase activity, and regulating some in vivo physiological responses. Pectins can influence starch digestion via multiple mechanisms simultaneously, in ways that are highly structure-dependent. Utilizing the multi-functionalities of pectin could provide more ways to design low glycemic-response food and while avoiding the unpalatable high viscosity in food by which is commonly caused by many other dietary fibers.

## 1. Introduction

Pectic polysaccharides, or pectins, form a family of polysaccharides in the cell walls of higher plants [1]. The major role of pectins in planta involves maintaining mechanical properties (for example, the rigidity of a stem) as a component of the cellulosic matrices which control the structure and properties of plant tissues [2]. The pectin contents in many fruits and vegetables range from 0.1% to 2.5% on a wet basis and a daily consumption of 30 g of pectins are recommended by the European Food Safety Authority for benefits such as reduction in post-prandial glycemic responses, maintenance of normal blood cholesterol concentrations and increase in satiety (the last leading to a reduction in energy intake) [3].

**Figure 1:**
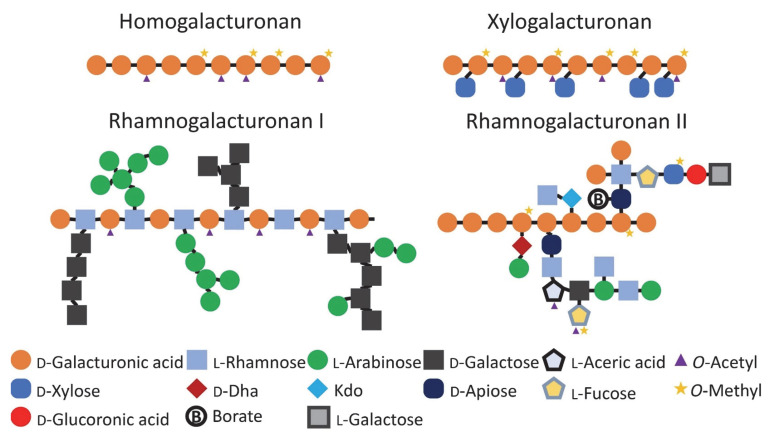
Schematic structural blocks of pectins.

Pectins have galacturonic acid (GalA) as their main component, and as many as 16 other monosaccharides as co-monomers [4]. A schematic representation of pectin structural components is shown in Figure 1. Complex pectins contain several constituent pectic blocks, namely homogalacturonan (HG), rhamnogalacturonan-I (RG-I), rhamnogalacturonan-II (RG-II) and xylogalacturonans (XGA); the last is present only in a few plants [1]. HG is a linear polysaccharide formed by (1→4)-α linked D-galactopyranosyluronic acid (Gal*p*A) residues. Carboxyl groups in HG are largely methyl-esterified when originally synthesized by the Golgi apparatus, and are then gradually de-esterified in vivo by pectinesterase during the growth of the plant. Based on the degree of esterification (DE), pectins can be divided into low methoxyl (LM) pectins (DE < 50%) and high methoxyl (HM) pectins (DE > 50%). The backbone of RG-I is the repeating disaccharide unit, [→4)- α -D-Gal*p*A-(1→2)- α -L-Rha*p*-(1→]. The side chains of RG-I are oligosaccharides composed mainly of neutral α-L-arabinofuranosyl (Ara*f*), β-D-galactopyranosyl (Gal*p*) or acidic β-D-glucuronosyl (Glc*p*A) residues [5,6]; these side chains are attached to the RG-I backbone via the C-4 of Rha*p*. Both rhamnogalacturonan-II and xylogalacturonans can be classified as substituted galacturonans [6], because their backbones are HG. 

Pectins from different sources, locations and growth conditions of plants and processing methods can differ in monosaccharide composition, molecular size/weight (both average size or weight, and size or weight distribution), degree of methyl esterification (DE) and degree of acetylation (DA), thus making the molecular structure of pectins very complex. Although sharing the same name, pectic polysaccharides with diverse compositions or structures can differ significantly in physicochemical and physiological properties as well as functions. For example, LM-pectins form a gel in the presence of divalent ions (mostly Ca^2+^) via coordination bonds [7], while HM-pectins gel via a combination of hydrogen bonds and hydrophobic interactions [8]. Pectins with higher DE values and lower molecular weights generally have a higher water solubility [2,8]. These examples show that, when studying a pectin’s effects, one should pay special attention to the molecular structure of that pectin and the status of pectin’s chemical environment (pH values, ionic strength, etc.). 

Starch provides at least half of the average daily caloric intake for most people world-wide [9]. After ingestion, starch is mainly hydrolyzed by α-amylases and intestinal brush border enzymes (i.e., sucrase, maltase and isomaltase) to glucose, which is absorbed in the intestine, leading to an increase in postprandial blood glucose levels [10]. A positive relationship has been proven between postprandial glycaemia level and obesity [11] and type II diabetes [12,13,14,15,16]. These findings indicate that controlling starch digestion is a promising approach to the prevention and management of these diseases. The digestibility of starch in food is mainly affected by the characteristics of the starch (e.g., molecular structure), the physicochemical conditions in the food matrix, the food processing method and the presence of other food components such as dietary fibers [17,18]. Several reviews have summarized the effects and mechanisms whereby dietary fiber can regulate the metabolism of carbohydrates in food to result in a healthier status [10,19,20,21,22]. 

Pectin has been found to regulate starch digestion, compared to other dietary fibers, pectins have some advantages in regulating starch digestion, including (1) ubiquity in food and wide applications in food industry, (2) reliable safety, (3) abundant health benefits, and (4) versatile molecular structures with the potential for tailor-made properties. With these advantages, people have studied pectin’s effects on starch in vitro digestion and in vivo utilization for decades, and from human subjects to a range of animals. 

However, there is no recent review summarizing pectin’s effects on starch digestion (i.e., passage of amylolysis products through the gastrointestinal (GI) tract, absorption of glucose and the resulting postprandial blood glucose level), making the findings in this field difficult to integrate. Additionally, many previous studies ignored the importance of pectin’s structure-property relationships, leading to ambiguous or misleading conclusions. To help solve these problems, in this review we discuss how pectin affects the in vivo utilization of starchy food. 

Generally, pectins regulate the digestion of starch by any or all of (1) inducing physicochemical changes in digesta, (2) inhibiting enzyme activities of amylases, (3) interacting with starch substrates, (4) being structural components in cell walls and (5) causing a series of physiological responses. The review concludes with discussion of the characteristics of pectins as modulators of starch digestion, some concluding remarks and suggestions on future perspectives. 

## 2. Effects of Pectin on In Vivo Starch Utilization

**Figure 2:**
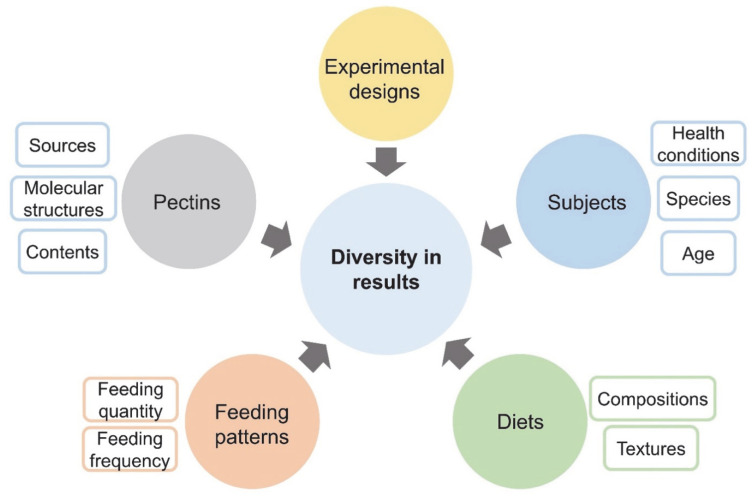
Experimental factors that may vary pectin’s effects on starch utilization.

The results obtained from in vivo investigations provide additional information as to how pectin ingestion affects physiological responses of humans and other animals. Pectin’s in vivo effects are complicated by the inherent variability in animal experiments (Figure 2), for example species of subjects, design of experiments procedures, measurement methods, selection and quantity of pectins, and composition of diets.

### 2.1. Human Studies

It is well accepted that a high fasting blood-glucose level (fBGL) is seen as a good indicator of diabetes, and the postprandial blood glucose level (pBGL) change is seen as a good measure of the metabolic processes that underlie type 2 diabetes [23]. Therefore, maintaining these glycemic levels in proper ranges is of great health interest. Ingestion of pectins, as a food additive or an ingredient in whole food, can show regulatory effects on postprandial blood-glucose level, fasting blood glucose level and in vivo digestion of starchy foods in human beings and other animals. Information from some such studies are shown in Table 1 and Table 2. 

In the 1970s, Jenkins and co-workers [24] conducted a series of experiments exploring the metabolic effects of dietary fibers on regulating postprandial blood-sugar and insulin levels. They firstly studied the effects of pectin and guar gum’s addition to food on the pBGL of 11 diabetic volunteers and found this fiber supplementation produced an overall decrease in postprandial blood-glucose concentration. For insulin-independent volunteers, the rise of pBGL from 15 to 90 min was halved, and a reduction of more than 36% in the mean rise of blood glucose from 45 to 180 min was found in the insulin-dependent volunteers The authors suggested the beneficial effects may arise from the slowed gastric emptying or from limited diffusion of food components in the gastrointestinal tract. 

The positive effect of pectins as a food additive was further confirmed with healthy human volunteers [25]. The results showed that the average pBGL was significantly lowered from 15 min after pectin was ingested and the average serum insulin concentration was also significantly lowered compared to the control from 15 to 45 min after measurement. It was also found that the combination use of pectin and guar gum in bread significantly reduced both volunteers’ pBGL and serum insulin concentrations, and the decrease was greater than that with pectin alone. 

It was also found by Jenkins and co-workers [28] that pectin in a test meal could flatten the pBGL, rather than simply decreasing it. Nine post-gastric surgery volunteer patients who also suffered from hypoglycemia were given 50 g of glucose with and without 14.5 g of pectin, and the results showed that pBGL values from pectin-containing were significantly below the controls at 15 and 30 min, and significantly above the controls at 90 and 120 min. This flattening effect avoided the hypoglycemia and sustained the energy homeostasis of the volunteers; the changes were ascribed to a slowed diffusion and absorption in the GI tract caused by the increased viscosity of digesta. 

The positive correlation between the food (chyme) viscosity induced by pectin and other DFs, and the average decrease in the maximum rise as well as the area under curve (AUC) in blood glucose concentration was confirmed in a subsequent work [27]. This supported the hypothesis proposed previously [24] that pectin could limit the diffusion of food in the GI tract, as pectin delayed the food’s mouth-to-caecum transit time by 15 min 

Holt et al. (1979) further confirmed pectin could delay the gastric emptying (GE). The authors found (1) when taking pectin and glucose together, the BGLs of volunteers were significantly reduced, and (2) the ingestion of pectin induced fewer gastric contents being emptied in 30 min and significantly longer gastric emptying half-times [26]. The long-term ingestion of pectin could also affect the GE: the GE half-time of 13 healthy adult volunteers was prolonged approximately twofold after pectin supplementation (*p* < 0.005) and returned to normal 3 weeks after discontinuing pectin supplementation [36]. 

Jones and co-workers [30] studied the effects of isolated soybean hull pectin on blood glucose and insulin responses in healthy male subjects. The results showed that the ingestion of soybean pectin decreased the pBGL, insulin concentrations and mean glucose incremental AUC compared to the control groups, although only the last of these was statistically significant. The analysis based on BMI results showed pectin induced a greater lowering of glucose and insulin response in subjects with normal body weight than in overweight and in obese subjects [30]. In addition, microbiota sequencing demonstrated an increase in the ratio of *Bacteroidetes* to *Firmicutes* in normal weight subjects compared to overweight subjects.

Although in many cases the addition of pectin to food showed regulating effects on BGL, its effects were not always favorable. Many factors, such as dosage and composition of other staple foods, could influence or mask the effects of pectins. This is because pectin is usually used only as a food additive, and, to avoid unpalatability, its content in food should not be dominant; therefore, when the starch is highly susceptible to amylase or the pectin concentration is rather low, there would be no significant impact from added pectin on starch digestibility. Nevertheless, pectin in a food may still affect the in vivo physiological responses to that food, without influencing the BGL. Two studies [34,35] have shown how a one-time ingestion of pectin as a food additive by healthy volunteers affected the digestion and uptake of food. Both studies found that a pectin-enriched meal caused delayed GE in volunteers but no significant differences in postprandial BGL compared to the control groups. 

Some other experiments focusing on the long-term ingestion of pectin by healthy [36] and unhealthy [37] volunteers showed that pectin did not change the postprandial BGL. Both studies showed that the pectins that did not affect BGL were not in a high dosage at any one time. Shimoyama et al. presented some unexpected results [33]. They found that the average GE coefficient, the rise of postprandial BGL and the insulin level were all increased by the intake of a high-viscosity pectin. The authors suggested these results may come from differences in measurement methods, test meals and subjects. 

### 2.2. Rodent Studies

#### 2.2.1. Studies on Commercially Available Pectin Samples

Using animals as subjects gives researchers more technical choices and flexibility than using human subjects, thus yielding more mechanistic and interesting results. Sánchez and co-workers added HM-pectin and β-glucan to the feed of obese rats and found that diets with both fibers reduced body weight, total cholesterol and triglycerides in Zucker fatty rats, while the pectin-enriched diet significantly decreased the fBGL and plasma insulin concentrations, showing pectin’s potential use in prevention against some of cardiovascular risk factors related to metabolic syndrome [40].

A comparative study on several DFs linked pectin’s effects on in vitro starch digestion to its influence in rat’s in vivo pBGL [39]. Pectin decreased the in vitro digestibility of gelatinized potato starch. As to in vivo studies, rats that had been fasted overnight were gavaged with gelatinized potato-starch suspensions with and without pectins, and results showed that pectin could significantly reduce pBGL of rats at 60 min after ingestion. Two correlations between in vitro and in vivo parameters were also found: resistant starch content was negatively correlated to the area under the glucose response curve, and the content of RDS positively correlated to the BGL 30 min after ingestion. This is potentially a useful result, because pectin’s in vivo and in vitro effects could be different under various conditions, and any difference may yield mechanistic information. 

Khramova et al. [38] elucidated how the gelling status of a homogalacturonan-like LM-apple pectin can influence the rheological properties of gastric digesta and the glycemic increase in mice. After administration to mice, pectin alone only formed viscous fluid in the mice’s stomachs, while pectin together with Ca^2+^ supplements under in vivo conditions formed a gel bolus. Both forms of pectin flattened the pBGL curve favorably; pectin itself reduced the BGL in the first 30 min of the postprandial period, while pectin-Ca^2+^ inhibited the glycemic response over a longer period. The presence of both forms of pectin significantly increased the viscosity of gastric digesta, but with different patterns. This shows that the physicochemical status of pectin supplements in the GI tract largely relates to its influence on digestion process. These researchers suggested that LM-pectin cross-linked with Ca^2+^ presented better effects on reducing starch digestion. However, these findings are not general, as results may be different from case to case, based on the variables in Figure 2. 

In most of the studies discussed above, only one type of pectin was used. To compare the glucose absorption inhibition of pectins with pectins’ DE values, Kim [43] tested both LM-pectin and HM-pectin for jejunal and ileal perfusions in rats. HM-pectin and LM-pectin significantly reduced the glucose absorption in both. Unsurprisingly, HM-pectin and LM-pectin increased the relative viscosities by more than a factor of 10 compared with the control, and both pectins could inhibit glucose absorption in the small intestine of rats. It was speculated that the inhibition of glucose uptake was caused by an increase in the mucosal unstirred layer thickness. 

#### 2.2.2. Isolated Pectin Samples from Natural Sources

Together with commercially available pectins, pectic polysaccharides extracted from natural sources, especially those that are common in food or herbal medicines, also exhibited decreasing BLG and anti-diabetic effects in rats [41,50]. A series of polysaccharides extracted from puerh tea (PTPS), with the principle constituent pectin having a notably high content of uronic acid, were found to have potent α-glucosidase inhibitory ability and significant postprandial anti-hyperglycemia ability in diabetic mice [42]. The researchers also found the PTPS from a longer aging time sample showed a higher content of uronic acid, which was positively correlated to its antioxidant and α-glycosidase inhibitory activities. Correspondingly, the PTPS with longer aging time also exhibited a greater decrease in the postprandial glycemic response of diabetic mice after a starch meal [42].

Recent researches in pectic polysaccharides extracted from natural food and medicinal plants also concentrated on looking for novel biochemical mechanisms for pectin’s hypoglycemic effects. Wu et al. [45] extracted a pectic polysaccharide (FPLP) containing hexenuronic acid from the fruits of *Ficus pumila* L. and found FPLP significantly reduced the fBGL and serum insulin of *db*/*db* mice in a dose-dependent manner after four weeks’ administration. FPLP is a typical HG-type LM-pectin with branched-chain hexenuronic acids. The authors suggested that the anti-hyperglycemic effect of FPLF comes from its improvement in hepatic glycogen metabolism in *db*/*db* mice, because FPLF (1) activates the IRS-1/PI3K/Akt/GSK3β/GS insulin signaling pathway and AMPK/GSK3β/GS signaling pathway and (2) regulates glucokinase, phosphoenolpyruvate carboxykinase and glucose-6-phosphatase expressions involved in hepatic glycogenesis and glycogenolysis. 

Yang and co-workers [47] extracted and fractionated several pectic polysaccharides from the mushroom *Gomphidiaceae rutilus*. They found that the total polysaccharide (denoted AGRP) and its neutral fraction (AGRP-N) reduced the BGL of *ob*/*ob* mice after four weeks’ oral administration, while the acidic fraction (AGRP-A) did not have this effect. The hypoglycemic effect of AGRP and AGRP-N may arise from enhancing lipolysis and autophagy to inhibit lipid accumulation in the liver, resulting in increased insulin sensitivity. 

*Panax ginseng* C. A. Meyer (ginseng) is a common traditional herbal medicine and food supplement with many health benefits [51]. Extracted pectic polysaccharide fractions from one ginseng sample [44] or several ginseng products [46] all exhibited a decreasing fBGL effect on diabetic mice. The latter researchers isolated pectic polysaccharides from heat-treated ginsengs and found pectic polysaccharides from ginsengs heated to higher temperature had better hypoglycemic effects, which may be because of the stronger antioxidant activity of the pectic polysaccharides [46].

### 2.3. Consumption of Pectin-Rich Whole Food and the Blood-Glucose Responses 

Several studies have explored the relationship between consumption of whole foods with high pectin content (without artificial addition of pectin) and the blood glucose response in human and rats [31,32,48]. Unlike what has been seen with extracted or commercial pectins, the investigation on whole food that is consumed as normal food or feed excluded some effects that may only exist in pure fibers (for example, excessively high viscosity or low palatability), and thus should yield a more relevant reflection of pectin’s effects in food. Nevertheless, pectin’s effects may vary with changes in composition and complexity of food. The same pectin-enriched test meal might have different effects in diabetic and healthy volunteers [31]. It was found that a high-fiber meal lowered the increase in postprandial BGL of diabetic but not of healthy volunteers; compared to low fiber meal intakes, high fiber meals also slowed the pBGL and changed the digestion rates of both diabetic and healthy volunteers. Additionally, whole foods with high contents of pectin, such as fruit by-products and yellow passionfruit peel flour, showed decreasing pBGL and reducing fasting blood glucose level effects on rats and on diabetic patients, respectively [32,48]. 

## 3. Mechanisms of Effects of Pectic Polysaccharides on Starch Digestion

**Figure 3:**
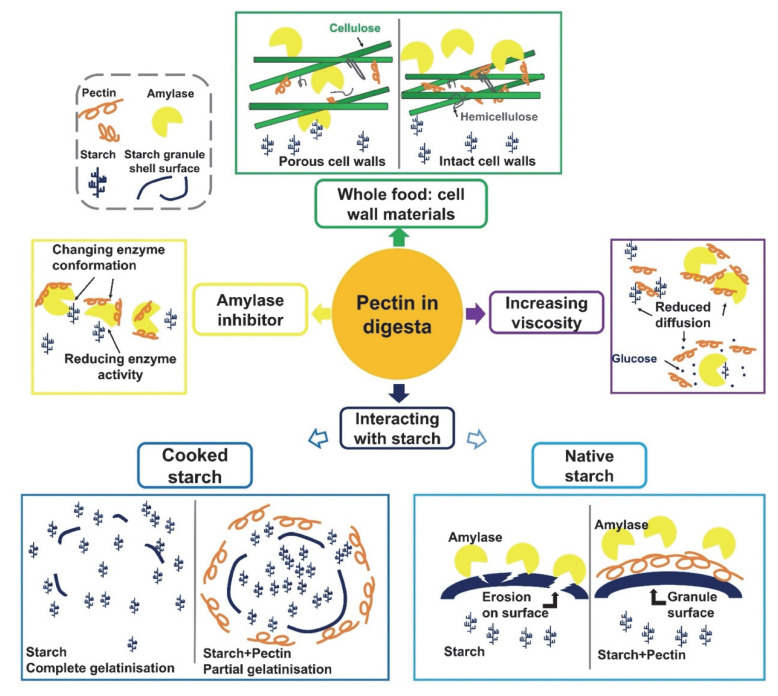
Schematic figure of pectin’s functions and mechanisms in regulating starch digestion process under in vitro conditions.

**Figure 4:**
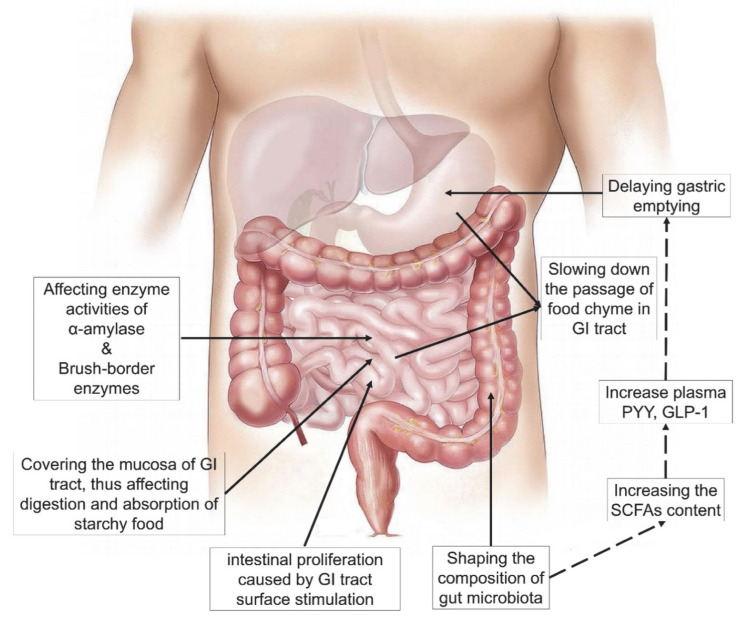
A summary of pectin’s regulating mechanisms in in vivo starch digestion and blood glucose level. Part of the graphic from https://pixabay.com/ (copyright-free, accessed on 2 June 2021).

As summarized in the previous section, pectin can affect in vivo digestion and absorption of starches, which are widely considered as beneficial for health. From the studies mentioned above, pectins with various origins and molecular structures have been tested in a large number of experiments, and have exhibited different effects on starch digestion and BGL regulation. The mechanisms underlying pectin’s effects are discussed in this section and are illustrated in Figure 3 and Figure 4, based on in vitro and in vivo conditions. 

### 3.1. Physicochemical Changes Caused by Pectin in Digesta

When dissolved in solution, hydrophilic chains of pectin molecules will hydrate and entangle with each other to form complex networks [52], which macroscopically will increase the solution viscosity. The physicochemical environments of in vivo digesta are complex because of (1) diverse chemical compounds (food components, enzymes, bile salts, etc.) in the GI tract, (2) variations in pH values and salt compositions in different phases of the GI tract, and (3) physiological responses stimulated by the ingestion of nutrients. The environments of digesta during passage through the GI tract are dynamic and complex. For instance, an in vivo experiment has shown that pectin does not form gel in the stomach at the beginning of ingestion because of unsuitable pH, but 2 h after ingestion, the stomach pH becomes optimal for gelling by gastric secretion [34]. 

Numerous in vivo investigations [26,27,28,39,53] have confirmed that the addition of pectin can increase the viscosity of digesta. Consequently, during passage through the GI tract, the blending of digesta, interaction between enzymes and substrates, diffusion of substrates and digestive enzymes [28,48,54] and the movement of chyme [29,53,55] are all slowed down, resulting in less digestion of starch and less nutrient absorption. It has been suggested that digesta mixtures could form gels because of pectin, which also leads to slowed movement through the GI tract [34,38,56].

### 3.2. Influence of Pectin on Amylase Activity 

Starch is digested sequentially in humans by salivary and pancreatic α-amylase, and by brush-border enzymes, specifically mucosal maltase–glucoamylase and sucrase–isomaltase. The end-product, glucose, is transported via enterocytes into the bloodstream. Inhibiting the activity of amylases has long been used as an effective approach to control postprandial blood glucose level and in vivo starch digestion [23,57]. Thus, finding more amylase inhibitors, especially those found in normal food, is of ongoing interest. 

Many researchers have studied the interaction between pectins and amylases; some of the results are as follows. Firstly, pectin can affect amylase activity under both in vitro [58,59] and in vivo [54,60,61] conditions. Secondly, pectins are able to present not only inhibitory but also enhancing [62,63] effects on amylase activity. Thirdly, they can influence many varieties of amylases, or amylase in different locations. For example, jejunum mucosal lactase and sucrase [60], α-amylase from the small intestine [62] and caecal amylase [63]. Lastly, they are effective in many species (e.g., humans [61], pigs [64], rats [55], and geese [54]). The diverse reported in vivo effects are influenced by many experimental factors, such as subjects, diet conditions, pectin samples, feeding patterns, experimental methods and analysis methods. Indeed, the measured changes in amylase activity may not deliver an altered in vivo digestion behavior, glucose uptake or growth performance of subjects, but the changes in measured amylase activity certainly reflect a changed biochemical environment of the GI tract or digesta. 

Effects on amylase activity by the addition of pectin could be due to several reasons. Firstly, this could reflect the changed physicochemical properties of digesta, for instance, increasing the viscosity of chyme [54,58], changing the pH values of digesta [58], and forming a gelling network in digesta [62]. The presence of pectin in food can indirectly affect enzyme activities by changing the physicochemical environments of amylases. In addition, pectin could also slow down the movement of relevant molecules, as mentioned above, and digestion-relevant processes (for example, the separation of amylolysis products such as glucose, maltose and maltotriose) in the digesta. From the point of view of thermodynamics, a higher local concentration of reaction products is not favorable for the reaction to proceed in the forward direction, although this is only relevant if the system is close to thermodynamic equilibrium.

Secondly, some pectins or pectic polysaccharides are able to inhibit amylase activity by intermolecular interaction. Ref. [64] reported that the addition of pectin to corn-starch-based diets for pigs decreased the activity of amylase by direct interaction between pectin and amylase, but did not influence either pancreatic juice secretion or the activity of other enzymes. Espinal-Ruiz and co-workers (2014) [59] found the dominant association between pectin and amylase was a hydrophobic interaction, which mainly happens on the amylase surface, where there is a high content of hydrophobic amino-acid groups, rather than at the catalytic sites. Under their conditions, α-amylase was negatively charged, which caused electrostatic repulsion with LM-pectin and less inhibition by LM-pectin. These results showed the potential application of pectins as inhibitors of digestive enzymes.

Other interaction conditions may also exist. For example, Bai et al. found pectins with lower DE values had better α-amylase inhibitory effects in a pH 6.0 sodium acetate buffer [65]. They found a polygalacturonic acid sample containing no methyl-ester groups and with a small molecular size could significantly (1) reduce the enzyme activity of porcine pancreatic α-amylase compared to other pectin samples and (2) decrease initial in vitro digestion rates of corn starch. Further analysis by Lineweaver-Burk and Dixon plots suggested that PGA’s inhibition kinetics followed a non-competitive pattern. The interaction between PGA and amylase is probably electrostatic, because, in vitro, pK_a,pectin_ < pH < pI_,protein_, which satisfies the optimal electrostatic interaction condition. Another HM-pectin fraction, CP, significantly accelerated initial in vitro digestion rates of corn starch under the same conditions, which may be caused by (1) the pectin gel network expelling α-amylase into the soluble phase, thus causing a higher amylase activity in that phase [66] or the hydration of pectin reduced the available water, resulting in a net increase in amylase concentration in solution. These results demonstrate pectin’s effects on amylase activity is dependent on both its molecular structure and chemical environment.

Finally, pectins in the diet can affect amylase activity by changing the physiological morphology of the GI tract. The pectin in the diet could be hydrated and form a viscous network after ingestion, and thus put a sticky layer on the surface of the GI tract during its transportation, affecting the activity of amylases around mucosa. Forman and co-workers found that pectin in the diet increased the total enzyme activity of mucosal-scraping amylase in the small intestine of rats, with a significant increase in the volume of small intestinal contents and the weight of the mucosal scraping [67]. Thomsen et al. found that pectin in the diet lowered both lactase and sucrase levels, with stronger effects than other food additives [60]. They suggested that pectin in food leads to the differentiation of the enterocytes, probably caused by contact between pectin and enterocytes, as confirmed by the study finding more immature enterocytes compared with the other diet groups. 

### 3.3. The Interplay between Pectin and Starch Substrate

When dispersed in water, pectin powder will undergo wetting, swelling, hydration, dissolution (often under heating or cooking conditions) and gelation when the chemical environment is favorable [68]. During these processes, the interaction between pectin and starch in digesta could strongly affect the starch higher-level structure. For example, a number of studies have found that the presence of pectin in starch could significantly influence starch gelatinization and swelling [69,70,71,72,73]. The addition of pectin decreased the peak, breakdown and final viscosity of the starch, i.e., limiting starch gelatinization when pectin and starch are heated together, as pectin may (1) adhere to the surface to starch granule and (2) compete with starch for free water (Figure 3). For ungelatinized starch substrates mixed with pectins, a common hypothesis is that pectin could coat the starch granule and form a physical barrier which protects starch from amylase and consequently decrease its digestibility [39,70,71]. These characteristics could be considered when designing pectin-containing starchy foods with a lower digestibility. When producing foods with pectin additives under in vitro conditions, several approaches that can eventually affect the digestibility of starch are given in Figure 3. 

### 3.4. Pectin as a Cell Wall Material in Whole Food

Plant cell walls play a significant role in the bioavailability and bioaccessibility of macromolecules: for example, the starch entrapped in cell walls [74]. In a whole plant food, the digestibility of starch is closely related to the intactness of cell walls, because the intact structures with a lower level of porosity could restrict the diffusion of extracellular amylase into cell walls [75,76]. It is reasonable that the digestibility of isolated starch would be higher than that of the same starch within cell walls in the native grain [77].

Pectin as a major cell-wall material significantly affects the morphology of plant cell walls, adhesion between cells and the texture of tissue [78,79]; as a consequence, the quantity and quality of pectin in cell walls both affect the digestibility of intracellular starch. This was also confirmed by Bi et al., who showed that, compared to native flour, flours without pectin have significantly higher digestibility under both raw and cooked conditions, suggesting pectin is crucial to the reduced flour digestibility caused by cell walls [77]. 

DE values of pectins have also been shown to be closely related to the digestibility of starch [80]. Thus, during the ripening of pumpkins, a series of de-esterification and depolymerization degradations of pectins occurs [79], and the breakdown of pectins leads to changes in the rigidity and porosity of the cell wall polysaccharide network, which increases the susceptibility of starch to amylase [80]. 

### 3.5. In Vivo Physiological Regulation Induced by Pectin Ingestion

Dietary fibers in food can induce a series of in vivo physiological responses related to starch digestion and glycaemia increase [10,81,82]. Pectin tends to blend with other food ingredients in the viscous bolus as it moves along the digestive tract and finally is fermented in the intestine. During this process, pectins can cause several interrelated physiological effects, such as slowing down GE, regulating the release of hormones, inhibiting nutrient absorption by changing the histology of the GI tract and shaping the population of colonic microbiota. There can be synergistic effects of these physiological responses, adding to the reasons for pectin’s in vivo effects, such as BGL regulation, increased satiety, and body weight management. In general, a single meal with a high amount of pectin will cause a delayed GE because of having an increased viscosity, which would prevent a rapid absorption of glucose by intestinal mucosa. On the other hand, a long-term ingestion of pectin-containing diets can lead to (1) histological changes in intestinal mucosal tissue with impaired glucose absorption ability or less amylase activity, (2) changes in hormonal responses, (3) systematic delay of GE and (4) combinations of these mechanisms [36]. The details of these are discussed below. 

#### 3.5.1. Gastric Emptying (GE)

GE is closely related to postprandial blood glucose level, because the stomach controls the provision of food load to the intestines, where food is digested and absorbed. The rate of GE and blood glucose level interact with each other so as to provide energy homeostasis [83]. Pectin after hydration would usually increase the viscosity of the food bolus and chyme, trap food ingredients and nutrients and slow down their transit through the GI tract. In studies using human subjects, a one-time ingestion of pectin in food was found to significantly prolong GE time [26,84] or delay the later phase of GE [34,35] in both healthy and obese subjects. The difference in delayed phases may be caused by the timing of formation of the pectin gel; pectin can only affect GE after forming viscous gel networks. This relationship between pectin’s viscosity and GE was shown in research using mice [38]: it was found that (1) ingestion of pectin significantly increased the viscosity of gastric digesta and delayed GE, and (2) pectin networks with higher gelling capacity showed better regulation of BGL and delayed GE. By comparison, the long-term ingestion of pectin could not only lead to a significantly viscosity-related delayed GE in healthy adult volunteers but also induce adaptive changes in the intestinal mucosa [36]. The delayed GE is reversible, as the symptoms disappeared within 3 weeks after stopping pectin supplementation. In addition, the pectin supplementation did not affect intestinal glucose, lysine level, water absorption, and plasma glucose or hormone responses. Besides delaying GE, it was also found that pectin dissolved in a liquid test meal with high viscosity could also accelerate GE [33]. The test meal (low viscosity, and rather high calorie content, fat content and volume) without pectin went rapidly into the duodenum and caused a duodenal brake, which inhibited GE compared to the test meal with pectin, which was viscous and also resulted in a duodenal brake, but much later. 

#### 3.5.2. Hormones

Ingested food could stimulate the GI tract to release hormones from enteroendocrine cells because of mechanical (such as inducing stomach distension) and chemical properties (for example, caloric density) [85]. Gastrointestinal hormones play an important role in short-term regulation of food intake and energy balance [10]. High blood-plasma concentrations of these hormones would inhibit GE and increase satiety, which could delay or suspend intestinal digestion process. In a series of rat studies targeting effects of dietary fibers (especially pectin) on hormone secretion, food intake and satiety, the authors found that the presence of these dietary fibers (1) increased the contents of plasma peptide tyrosine tyrosine (PYY) and glucagon-like peptide-1 (GLP-1) at least twofold and (2) decreased food intake, weight gain and adiposity. They also suggested that the presence of soluble fiber was more critical than its source [86,87,88]. 

#### 3.5.3. Histology of the Gastrointestinal Tract

The sustained ingestion of pectin can lead to histological and morphological changes in the GI tract. This results in increases in the weight or length of the pancreas [54], small intestine [54,55,87,88,89] and large intestine [54,55,87,88]. Longer intake time, higher intake content and higher viscosity of pectins positively lead to the weight increase. The viscous pectic gels could contact and stimulate the surfaces of the stomach and intestines and affect their normal development and functioning. Consequently, pectin could cause both compensation development or muscle hypertrophy of gut tissues and chyme with high viscosity that sticks on the GI tract, which are the two main reasons for the increased weight and length of the GI tract. Intestinal proliferation has been considered as a reason for the increase in food digestion caused by pectin, because the total number of L-cells (producing PYY and GLP-1) may also increase along the GI tract [87]. Gut morphology could also be affected [60,87]. The presence of pectin in the gut was also found to impair absorption of nutrients, including glucose. Various studies have suggested that the increased gut viscosity brought about by pectin could thicken [53,90], or increase the resistance of, the mucosal unstirred layer [43], which eventually would reduce glucose absorption. Finally, pectin in the diet could also soften and increase the output and moisture of the feces, easing constipation and hemorrhoids [32,50].

#### 3.5.4. Gut Microbiota and Short-Chain Fatty Acids

The importance of gut microbiota in digestion has been extensively studied [91,92,93]. Pectins with higher solubility are usually easily fermented by microbes in the distal sections of the GI tract. During fermentation, the presence of pectin contributes to shaping the composition of gut microbiota, such as enhancing the diversity [94,95] or promoting the growth of probiotic intestinal bacteria [96,97]. The fermentation generates short-chain fatty acids (SCFAs) as products, including acetate, propionate, butyrate, succinate and lactate, which are beneficial to colonic health, cancer and inflammation inhibition, appetite regulation and energy homeostasis [88]. Pectin in the diet could significantly increase the SCFA concentrations in the caecum and colon [30,56,86,88], more so than some other DFs (cellulose, fructo-oligosaccharide, oat β-glucan) and proteins, perhaps because of pectin’s higher solubility and fermentability. Pectin may also impair the intestinal digestion of starch, letting more starch escape amylolysis to be finally fermented in the GI tract, which is another reason for the increased SCFA content [30,98]. SCFAs could promote the secretion of digestive hormones (such as PYY and GLP-1) [88,99,100,101] and mucous growth and epithelial cell proliferation in the intestine [102,103]. A high content of SCFAs produced by pectin may also decrease the pH of the GI tract, thus inhibiting the growth of pathogenic bacteria [48]. 

## 4. The Characteristics and Future Perspectives of Pectin’s Effects on Starch Digestion

From the above mentioned studies about pectins as nutritional additives in food targeted to regulate starch digestion, we can find that the addition of pectin to digesta can normally regulate the in vivo BGL of subjects from different species and health conditions. Pectins share some similarities to other DF nutritional additives: for example (1) causing an increase in viscosity is one of the major reasons to their changes and (2) the effects are normally concentration-dependent. 

Nevertheless, pectins also exhibit unique characteristics: some functional properties are determined by their complex and specific molecular structures. Many publications have clearly confirmed that pectin substrates with various structural parameters (DE values, monosaccharide compositions and molecular sizes) exhibit different extents of retardation of starch digestion. In addition, it was also observed that starch-containing digesta with pectin could have ideal digestibility [29,40] and are less dependent on viscosity [39] compared to other DFs (for example, pullulan, xanthan gum, guar gum and konjac glucomannan), suggesting pectic fractions take effects via more than one approaches simultaneously. The structure-based multi-functionality makes pectin stand out from other nutritional additive fibers, because a high viscosity in food can easily cause some unfavorable effects, including vomiting [26], abdominal discomfort [26,32], flatus [24] and diarrhea [104], as well as low food palatability [25,32,49,105]. Increasing the viscosity, either differing in concentration or molecular structure, is pectin’s dominant effect on digesta and a major reason for its regulatory effects. However, compared to other dietary fibers, effects of pectins do not simply rely on increasing viscosity of food or chyme, thus showing the multiple-functional advantages of pectins. Any adverse effects can be avoided or reduced by using pectins with other significant functional properties in food. For example, pectins with certain structural characteristics show significant digestion regulation ability without causing a high viscosity, although more in-depth studies are necessary in their practical applications [80]. The multiple functionality of pectins also gives flexibility in food production: e.g., pectins can affect both starch gelatinization and retrogradation, indicating that pectins can be added to starchy food either before or after thermal treatment, while both processes are able to regulate the digestibility of starchy foods. 

The structure-based multi-functionality of pectins in affecting starch digestion showed great potentials in future applications. Pectic fractions with different molecular structure, although generically classified as pectins, could result in very different properties under the same conditions. One disadvantage in many previous studies has been the lack of structural characterization of the pectin samples used, meaning that the results obtained from these studies cannot be used to understand which property or molecular domain of pectin functions were determinant. Although pectin’s effects on starch digestion and regulation on BGL have often been studied, the absence of extensive structure-property relationships leaves much to be done. Comparisons on functions from structurally different pectins are needed. To achieve that goal, firstly, structural information of the selected pectin sample should be obtained. Some basic parameters, like DE, monosaccharide composition and molecular sizes are essential, while more detailed data including acetylation degree, sugar linkage composition and side-chain composition would be beneficial. The experimental methods for investigating pectin’s effects on starch digestion could be standardized. For in vitro experiments, impracticable conditions (e.g., very low enzyme quantity or unacceptably high food digesta viscosity) should be replaced by methods (both apparatus, e.g., [106] and protocols [107,108,109,110,111]) which better simulate in vivo conditions. After obtaining some important structure-property (amylase inhibitory and starch granule binding, etc.) relationships, pectins with ideal molecular structures from either isolation from natural sources or chemical/enzymatic modifications could be further used for in vivo tests and subsequent use in foods.

## 5. Conclusions

Regulation of the digestion process of starch in food is an effective and promising way to control postprandial blood-glucose levels, which are closely related to the prevention and treatment of many pandemic health issues, such as obesity, hyperglycemia and type II diabetes. The presence of dietary fiber in food can significantly affect the digestibility of starch. Among the DFs, pectin is of particular interest, because of (1) its ubiquity in plant foods, (2) its versatile functions as an artificial food additive, (3) safety and reliability, and (4) various health benefits. Pectins have considerable complexity in molecular structure. Many researchers have studied the effects of pectin on starch digestion and the changes in in vivo blood glucose level brought by pectin in food, and most of these researches demonstrated that the presence of pectin could retard the digestion process or flatten the BGL. 

There are three major reasons for pectin’s decreasing effect on digestibility and BGL, as follows. (1) Pectin would normally increase the viscosity of digesta or food chyme, thus inducing a series of physicochemical and physiological changes; (2) pectin could interact with amylases and inhibit their activity; and (3) the ingestion and fermentation of pectin in intestines also regulate digestion-related in vivo physiological responses. In vivo, an increased viscosity of chyme or food bolus could prolong the transit time of food in the GI tract, which would delay gastric emptying [26,34,35,36]. Increased intraluminal viscosity also slows the absorption of nutrients in the intestine and affects the production of digestion-related hormones. Pectin gel that sticks on the surface of the GI tract can also lead to histological or morphological changes in the tract, affecting the intestinal absorption ability and amylase activities. Besides influencing amylase activity by differentiating the growth of enterocytes, pectins have also been found to inhibit the activities of starch-digestion related enzymes. Both pectin and amylase have abundant functional groups and can thus interact with each other via non-covalent intermolecular interactions. The fermentation of pectin in the intestine could also contribute to BGL regulation. Pectin, being an indigestible fiber, can only be fermented by gut microbiota, and thus the presence of pectin could (1) physically stimulate the distal intestines, (2) influence the growth and balance of microbiota and (3) promote the production of SCFAs during fermentation. The SCFAs partially influence the content of plasma digestive hormones, which are responsible for appetite, satiety and GE, and thus indirectly influence BGL fluctuations and in vivo digestion of food. 

## Figures and Tables

**Table 1:** Effects and mechanisms of pectin on postprandial and fasting blood glucose levels with human subjects.

Subject	Reference	Effect	Mechanisms	Term of Ingestion	Pectin Information	Pectin Dosage or Concentration	Diet
Human Experiments							
8 non-insulin dependent and 3 insulin-dependent diabetic patients	[24]	↓ pBGL *	-	single intake	NA	16 g guar and 10 g pectin	16 g guar and 10 g pectin to the control meal containing 106 g carbohydrate
healthy human: age 19–33	[25]	↓ pBGL	-	single intake	NA	10 pectin in 80 g marmalade; 33 mg/mL	70 g white bread + 16 g butter + 80 g marmalade + 300 mL of tea (43 g milk)
healthy human: age 25–32	[26]	↓ pBGL	increase viscosity; delay gastric emptying	single intake	NA	72.5 mg/mL; 5.5% wt	glucose: 50 g in 200 mL
11 human volunteers: age 20–40	[27]	↓ pBGL	increase viscosity; slow absorption; prolong transit time	single intake	NA	14.5 g pectin; ~36 mg/mL	50 g glucose, 25 g xylose, 15 g lactulose, and 40 g Pure Lemon Juice in 400 mL of water
9 post-gastric surgery patients	[28]	flattening pBGL	increase viscosity; Prolonged absorption	single intake	HM-pectin	14.5 g HM pectin; 36.25 mg/mL	50 g glucose + 400 mL water + 40 g of pure lemon juice + 14.5 g of HM-pectin
healthy male: age 22–26, BMI 20	[29]	↓ pBGL	increase viscosity	single intake	HM-pectin DE 68%; 140 kDa	30 g; 10% wt%	50 g glucose in 20% solution (Aguettant, France) alone or mixed with 30 g pectin
30 healthy males: age 18–45	[30]	↓ pBGL	-	single intake	soybean pectin	10 g; 16.7 mg/mL	10 g pectin + 50 g glucose + 600 mL water
8 diabetes: average age 54, BMI 28; 8 healthy: average age 21, BMI 21.	[31]	↓ pBGL	by prolonging intestinal digestion and/or absorption	single intake	natural pectin in apple and whole grain bread	high fiber group-12 g, 12% (wt%); low fiber group-4 g, 7% (wt%)	Margarine milk cheese bread apple
43 diabetic patient volunteers: age 57–73, average weight 66.8 kg, average BMI 27.8	[32]	↓ fBGL	increase viscosity, (hypothesis)	long term intake	yellow passion fruit peel flour	about 6.3 g/day	30 g flour, equal to 17.4 g of total fiber (6.3 g of soluble fiber and 11.1 g of insoluble fiber),
11 healthy volunteers: age 22–35	[33]	↑ pBGL	accelerate gastric emptying	single intake	NA	90 g; 180 mg/mL	400 mL, 400 kcal; protein, 14.8 g; fat, 14.4 g; glucose, 52.8 g; dietary fiber, 4 g + inorganic salts
10 healthy humans	[34]	× pBGL	slowed gastric emptying	single intake	pectin in drink	2 g	2 eggs (60 g), 30 mL milk, 25 g butter, 2 slices of toast and 300 mL high-glucose drink 61.5 g carbohydrate. 655 kcal
10 healthy male: age 21–33, 56.8–73.2 kg, BMI 19.4–23.9	[35]	× pBGL	slowed gastric emptying	single intake	NA	5 g; 10 mg/mL	450 kcal energy and 500 mL; 70 g Maltodextrin + 5 g glucose + 9 g fat + 17 g protein
13 healthy adults: age 18–37	[36]	× pBGL	-	4 weeks	pectin baked in muffin	20 g/day	2400-calorie, 50% carbohydrate, 3 g crude fiber.
66 unhealthy human volunteers: age 30–65, 70–90 kg, BMI 25–32	[37]	× pBGL & fBGL	-	12 weeks	SBP	16 g/day; 40 mg/mL SBP (soluble fiber content, 76%)	was 40 g white bread, 40 g cucumber, 160 g orange juice and 2 dL drink

* ↓ represents down regulation; × represents having no effect; ↑ represents up regulation.

**Table 2:** Effects and mechanisms of pectin on postprandial and fasting blood glucose levels with animal subjects.

Subject	Reference	Effect	Mechanisms	Term of Ingestion	Pectin Information	Pectin Dosage or Concentration	Diet
Animal Experiments							
Swiss albino mice; 30 g	[38]	flattening pBGL *	form gel; increase viscosity; affect gastric emptying	single intake	LM-pectin with structural information	2% in solution	chow diet containing 5% lipid, 14% protein and 76% carbohydrate, including 5% dietary fiber (cellulose)
health male Sprague Dawley rats	[39]	↓ pBGL	inhibit enzyme digestion process	single intake	Citrus pectin (Sigma Aldrich P9135)	5% based on starch dry weight	1.8 g gelatinized potato starch + 90 mg pectin; 10 mL diet per kg of rat’s body weight
Zucker fatty rats; 260–275 g	[40]	↓ fBGL	form gel and increase viscosity	long term intake	HM 73% apple pectin	10% wt%	10% pectin + protein (14%), fat (4%), and carbohydrates (72%)
alloxan-induced diabetic rats	[41]	↓ fBGL	increase viscosity	5 days	passion fruit pectin	0.5–25 mg/kg orally	-
alloxan induced diabetes Male ICR mice; 20 g	[42]	↓ pBGL	amylase inhibitory (assumed)	single intake	puerh tea pectic polysaccharides	50 mg/kg of body weight	soluble starch (2 g/kg BW) alone
Male Sprague-Dawley rats	[43]	↓ Glucose absorption	increase viscosity; affect the unstirred layer in intestines	single intake	Citrus pectin; DE 30% and 90%	10 mg/mL	perfusions: pH 7.4 + pectin (10 g/L) + glucose 10 mmol/mL, etc.
Male ICR STZ induced diabetes mice; 6–8 weeks old; 24 g	[44]	↓ fBGL	anti-oxidation; stimulating increased insulin secretion	10 days	ginseng pectin	with WGP (50 mg per kg per day), WGPA (10 mg per kg per day) and WGPN (30 mg per kg per day)	-
Genetically diabetic C57BL/Ksj *db*/*db* male mice; 5 weeks	[45]	↓ fBGL	improving hepatic glycogen metabolism	4 weeks	extracted FPLP	100 mg kg^−1^ day^−1^ (DFL); FPLP 200 mg kg^−1^ day^−1^ (DFH)	-
ICR alloxan induced diabetic male mice; 20 g; 6–8 weeks	[46]	↓ fBGL	anti-oxidation	4 weeks	ginseng pectins	100 mg/kg of GPW, GPR and GPS,	-
Male C57BL/6 J and *db*/*db* mice	[47]	↓ pBGL	increased insulin sensitivity by inhibiting lipid accumulation in the liver	5 weeks	mushroom pectic polysaccharides	50 mg/kg	testing solution: glucose solution 2 g/kg; diet (60% cereals, 33% protein and 3% oil) and water
Male Wistar rats; 21 days	[48]	↓ pBGL	increase viscosity: slow diffusion and absorption	32 days	Apple pomace Orange bagasse Passion fruit peel	~2% wt in diet	Casein, sucrose, soybean oil, starch, cellulose and by-products
4 horses; 12 yrs; 642 kg	[49]	× pBGL	-	single intake	apple pectin	0.1 g/kg bodyweight	pellet containing 50% corn starch and 25% apple pectin

* ↓ represents down regulation; × represents having no effect.

## Data Availability

Available from the authors on request.
